# Construction of three-dimensional, homogeneous regenerative cartilage tissue based on the ECG-DBM complex

**DOI:** 10.3389/fbioe.2023.1252790

**Published:** 2023-09-25

**Authors:** Jingwen Liu, Feifan Chen, Daiying Song, Qixin Zhang, Peizhe Li, Zheng Ci, Wei Zhang, Guangdong Zhou

**Affiliations:** ^1^ Research Institute of Plastic Surgery, Wei Fang Medical College, Weifang, China; ^2^ Shanghai Key Laboratory of Tissue Engineering, Department of Plastic and Reconstructive Surgery, Shanghai 9th People’s Hospital, Shanghai Stem Cell Institute, Shanghai Jiao Tong University School of Medicine, Shanghai, China; ^3^ The Affiliated Taian City Central Hospital of Qingdao University, Taian, China; ^4^ Department of Geratology, Weifang People’s Hospital, Weifang, China; ^5^ Department of Thoracic Surgery, Shanghai Pulmonary Hospital, Tongji University School of Medicine Shanghai, Shanghai, China

**Keywords:** engineered cartilage gel, decalcified bone matrix, 3D cartilage regeneration, tissue engineering, shape retention

## Abstract

**Introduction:** The feasibility of using a steel decalcified bone matrix (DBM)-reinforced concrete engineered cartilage gel (ECG) model concept for *in vivo* cartilage regeneration has been demonstrated in preliminary experiments. However, the regenerated cartilage tissue contained an immature part in the center. The present study aimed to achieve more homogeneous regenerated cartilage based on the same model concept.

**Methods:** For this, we optimized the culture conditions for the engineered cartilage gel-decalcified bone matrix (ECG-DBM) complex based on the previous model and systematically compared the *in vitro* chondrogenic abilities of ECG in the cartilage slice and ECG-DBM complex states. We then compared the *in vivo* cartilage regeneration effects of the ECG-DBM complex with those of an equivalent volume of ECG and an equivalent ECG content.

**Results and discussion:** Significant increases in the DNA content and cartilage-specific matrix content were observed for the ECG-DBM complex compared with the ECG cartilage slice, suggesting that the DBM scaffold significantly improved the quality of ECG-derived cartilage regeneration *in vitro*. In the *in vivo* experiments, high-quality cartilage tissue was regenerated in all groups at 8 weeks, and the regenerated cartilage exhibited typical cartilage lacunae and cartilage-specific extracellular matrix deposition. Quantitative analysis revealed a higher chondrogenic efficiency in the ECG-DBM group. Specifically, the ECG-DBM complex achieved more homogeneous and stable regenerated cartilage than an equivalent volume of ECG and more mature regenerated cartilage than an equivalent ECG content. Compared with ECG overall, ECG-DBM had a more controllable shape, good morphology retention, moderate mechanical strength, and high cartilage regeneration efficiency. Further evaluation of the ECG-DBM complex after *in vitro* culture for 7 and 14 days confirmed that an extended *in vitro* preculture facilitated more homogeneous cartilage regeneration.

## 1 Introduction

Cartilage defects are clinically common, have a poor intrinsic self-healing ability due to their avascular and nerve-free nature, and are generally treated with autologous tissue grafts and biomaterial fillers (Williams et al., 2010; Huey et al., 2012; Rasheed et al., 2019). However, these treatment methods have inherent problems, including irreversible damage to the donor area and lack of biological function. Therefore, better methods for cartilage repair are urgently required. Tissue engineering techniques offer a promising approach to cartilage regeneration and repair because they can achieve a large amount of regenerated cartilage from a small piece of autologous cartilage through cell isolation and *in vitro* expansion (Armiento et al., 2018; Xia et al., 2018; Wang et al., 2020). Nevertheless, the construction of three-dimensional (3D) regenerated cartilage with a specific shape and appropriate mechanical strength for the repair of large cartilage defects remains a huge challenge (Xu et al., 2020; Chae et al., 2021). Previous studies demonstrated that cartilage sheets constructed without a scaffold, as well as engineered cartilage gel (ECG) prepared from these sheets, can regenerate high-quality cartilage after subcutaneous implantation (Schulze-Tanzil et al., 2002; Schuh et al., 2012; Xu et al., 2022a; Xu et al., 2022b). These procedures have several important advantages over direct inoculation of chondrocytes as seed cells onto scaffold materials for cartilage regeneration, including the lack of inflammatory response to a scaffold material, higher seed cell density, and regeneration of high-quality cartilage. However, the poor plasticity and low mechanical strength of ECG render it difficult to regenerate cartilage tissue with specific 3D structures and sufficient mechanical strength using ECG alone. Furthermore, the large size of ECG particles makes them unsuitable for implantation with conventional porous scaffold materials. Thus, the application of ECG alone is mainly limited to injectable micro-trauma repair (Ding et al., 2021; Xu et al., 2022a).

To solve the problem of constructing regenerated cartilage with a certain volume using ECG, a suitable biological scaffold material is urgently needed. Scaffolds are a core component of tissue engineering with critical roles in determining the cell location and directing matrix formation (Asghari et al., 2017; Chaudhuri et al., 2017; Xie et al., 2017). As a suitable scaffold material for construction of regenerated cartilage with a particular 3D morphology, the decalcified bone matrix (DBM) has superior biocompatibility and appropriate structural stability (Man et al., 2016). However, these beneficial features can easily be lost after cell implantation due to the large pore size and difficulty in controlling the structural homogeneity. DBM cannot be used directly for cartilage regeneration (Osch et al., 1998; Xu et al., 2017a). Interestingly, the larger pore size is able to address the problem of incorporating large ECG particles. Furthermore, the excellent biocompatibility and mechanical properties of DBM can compensate for the inability to regenerate a specific volume of cartilage using ECG alone. The study combined the characteristics of both ECG and DBM to develop a new method for engineered cartilage gel-decalcified bone matrix (ECG-DBM)-based cartilage regeneration. In addition, DBM has a 3D sponge-like structure with high porosity and an interconnected porous structure (Zhang et al., 2019). These features provide a broad internal surface area and sufficient space for ECG, and represent favorable conditions for chondrocyte formation and matrix production. Therefore, the composites constructed by combining ECG and DBM can achieve tissue-engineered cartilage with a certain shape and appropriate mechanical strength.

In this study, we also investigated whether the ECG-DBM complex can achieve superior regenerated cartilage quality and yield compared with an equivalent volume of ECG (EV) and an equivalent ECG content (EEV). Previous studies demonstrated that the cartilage regenerated from subcutaneously implanted ECG-DBM complexes contained inferior cartilage in the center, leading to a heterogeneous structure in the sample (Wu et al., 2010). One possible reason was the short *in vitro* culture cycle. Another possible reason was that the ECG-DBM complex had not yet formed mature cartilage-like tissue. Apoptosis of immature tissue is usually induced in the center when the peripheral cartilage gradually matures after subcutaneous implantation (Bonzani et al., 2006). Therefore, it is necessary to optimize the *in vitro* culture cycle to achieve more stable subcutaneous cartilage regeneration using ECG-DBM complex technology.

The present study aimed to further optimize the culture cycle of the ECG-DBM complex based on the previous model and compare the cartilage regeneration effects of the ECG-DBM complex with those of EV and EEV to clarify the cartilage regeneration potential of ECG-DBM complex technology. The study provides an ideal treatment method for related cartilage repair and regeneration challenges.

## 2 Materials and methods

### 2.1 Culture of chondrocytes and cartilage sheets

The study was approved by the Weifang Medical College Ethics Committee. New Zealand white rabbits (two to three months of age) were purchased from Shanghai Jiagan Experimental Animal Raising Farm (Shanghai, China). Ear cartilage was taken from the rabbits, cut into 1 × 1-mm^2^ pieces, added with 0.2% NB4 collagenase (Nordmark Biochemicals, Germany), and digested on a shaker at 37°C for 8 h. Isolated chondrocytes were collected and cultured in a chondrocyte proliferation medium, consisting of Dulbecco’s modified Eagle’s medium (DMEM; Gibco BRL, Grand Island, NY, United States) supplemented with 10% fetal bovine serum (Gibco BRL) and 1% antibiotic-antimycotic solution (Gibco BRL). The chondrocytes were passaged at >80% confluence and cultured to the second (P2) or third (P3) generation for use in subsequent experiments.

Cartilage sheets were prepared as previously described (Xue et al., 2018). Briefly, chondrocytes were collected, seeded into six-well plates at high density (1.5 × 10^6^ cells/cm^2^), and cultured in a chondrocyte proliferation medium for 3 days. The medium was then replaced with a chondrogenic induction medium, and the culture was continued to grow. The chondrogenic induction medium consisted of the DMEM basal medium supplemented with TGF-β1 (R&D Systems Inc., Minneapolis, MN, United States), 40 ng/mL dexamethasone (Sigma-Aldrich, St. Louis, MO, United States), 100 ng/mL IGF-I (R&D Systems Inc.), 1% insulin–transferrin–selenium–linoleic acid (ITS; ScienCell, CA, United States), and 1% antibiotic-antimycotic solution (Gibco BRL). After 4, 7, and 14 days of *in vitro* culture to allow chondrogenic induction, cartilage sheets with different maturity levels were used for subsequent experiments.

### 2.2 Preparation of ECG and ECG-DBM complexes

Cartilage sheets that had been cultured *in vitro* for 4, 7, and 14 days were minced into pieces and collected in a centrifuge tube without any further attenuation to prepare 4-d ECG, 7-d ECG, and 14-d ECG, respectively.

DBM frameworks (Daqing Bio Co. Ltd., Chongqing, China) were cut into cuboid constructs (length: 7 mm; width: 5 mm; thickness: 2.5 mm). All constructs were sterilized using ethylene oxide before use. The previously described 3-d ECG was collected using a 5-mL syringe and thoroughly mixed with a 1.5-fold volume of the chondrogenic medium. The 3-d ECG and DBM were then combined by centrifugation at 500 rpm for 1 min to complete the inoculation of ECG onto DBM. The ECG-DBM constructs were gently transferred into new six-well plates and incubated for 2 h before the addition of the cartilage induction medium. The constructs were collected after 4, 7, and 14 days of induction culture and used for subsequent experiments.

### 2.3 Biocompatibility assessment between ECG and DBM

#### 2.3.1 Scanning electron microscopy observation

After 4, 7, and 14 days of *in vitro* culture, the ECG-DBM complexes were washed with PBS and fixed with 0.05% glutaraldehyde. DBM and ECG-DBM complexes (*n* = 3 per group) were then dried by the critical point drying method. The samples were observed using a SEM microscope (XL-30; Philips, Amsterdam, the Netherlands) and photographed to assess the pore size distribution in the DBM constructs, the attachment of ECG, and the matrix synthesis (Xu et al., 2017b).

#### 2.3.2 Assessment of ECG adhesion and the proliferation efficiency on DBM

The ECG adhesion rate was determined by the ratio of the DNA content of ECG seeded into the DBM and the DNA content of the ECG-DBM constructs after culture for 24 h. The DNA contents of the samples (*n* = 5 per group) were quantified using the Quant-iT PicoGreen dsDNA assay (Invitrogen, United States), as described in this article (He et al., 2018). The proliferation efficiency of ECG on DBM was confirmed by the rate of change in the wet weight and volume of the ECG-DBM constructs during *in vitro* culture.

#### 2.3.3 Quantitative and mechanical analysis of regenerated tissues

After observation of the gross morphology, all samples (*n* = 5 per group) were weighed using an electronic balance. The volume of each sample (*n* = 5 per group) was measured using a water displacement method. The Young’s modulus of the regenerated cartilage was tested using a mechanical analyzer (Instron-5542; Instron, Canton, MA, United States). As previously described, the samples from different groups (*n* = 5 per group) were cut into 4-mm-diameter cylinders. A constant compressive strain at a speed of 0.5 mm/min was applied until failure of the sample (turning point on the force-displacement curve). Stress–strain curves were obtained from the first 40%. The Young’s modulus was calculated according to these stress–strain curves. The compressive strength was defined as the force when the sample underwent fragmentation.

### 2.4 Evaluation of *in vitro* cartilage regeneration performance of ECG and ECG-DBM complexes

#### 2.4.1 Histological and immunohistochemical analyses

After completion of the gross morphology observations and measurements, a portion of each sample was fixed in 4% paraformaldehyde, embedded in paraffin, sectioned at 5-μm thickness, and mounted on glass slides for histological and immunohistochemical analyses. The histology of the regenerated cartilage was evaluated by H&E staining and safranin O (SO) staining. Immunohistochemical analysis was conducted to evaluate the expression of type II collagen using a rabbit anti-human collagen monoclonal antibody (COL-2) and a horseradish peroxidase-conjugated anti-rabbit secondary antibody (Santa Cruz Biotechnology, Santa Cruz, CA, United States; 1:400 dilution in PBS), as previously described (Gatam et al., 2017).

#### 2.4.2 Quantitative biochemical analysis

The remaining samples were used for biochemical analyses. All samples (ECG, DBM, ECG-DBM complexes, and natural cartilage specimens) were weighed and minced. DNA, sulfated glycosaminoglycan (GAG), and collagen were quantified using previously established methods (Roberts et al., 2009; Wang et al., 2020; Chen et al., 2021b). Briefly, the GAG contents in the samples (*n* = 5 per group) were quantified using an Alcian blue method, the total collagen contents in the samples (*n* = 4 per group) were quantified using a hydroxyproline assay, and the DNA contents in the samples (*n* = 5 per group) were quantified using the Quant-iT PicoGreen dsDNA assay. For the quantitative analyses of the GAG and collagen contents in the ECG-DBM constructs, we avoided any influence of the GAG and collagen contents in the DBM itself by incubating the samples under the same conditions for 14 days. The specimens were collected at 4, 7, and 14 days, and the GAG and total collagen contents were measured and averaged. The mean values were used for comparisons in statistical analyses.

### 2.5 ECG and ECG-DBM complexes for subcutaneous cartilage regeneration in nude mice

*In vivo* 3D cartilage formation is an important criterion for determining whether a scaffold is suitable for cartilage engineering (Kim et al., 2016). To investigate the *in vivo* chondrogenic efficiency and optimal implantation time for ECG and the composite materials, we subcutaneously implanted ECG-DBM complexes cultured *in vitro* for 7 or 14 days into nude mice. As a control group, 7-d ECG and 14-d ECG were injected subcutaneously into the dorsum of nude mice at an equivalent volume of ECG and an equivalent ECG content to the ECG-DBM group, respectively. For these experiments, EV was the mean volume of ECG-DBM measured during *in vitro* culture, i.e., 100 μL at 7 days and 130 μL at 14 days, and EEV was the mean ECG volume inoculated into ECG-DBM, i.e., 70 μL at 7 days and 90 μL at 14 days. The samples were collected at 4 and 8 weeks after transplantation and subjected to gross morphological, histological, immunohistochemical, and quantitative assessments.

### 2.6 Statistical analysis

Data were expressed as mean ± standard deviation. A *t*-test was used to compare the differences between groups, and values of *p* < 0.05 were considered statistically significant. SPSS 23 software was used for all statistical analyses.

## 3 Results

### 3.1 Preparation of ECG and ECG-DBM constructs

**FIGURE 1:**
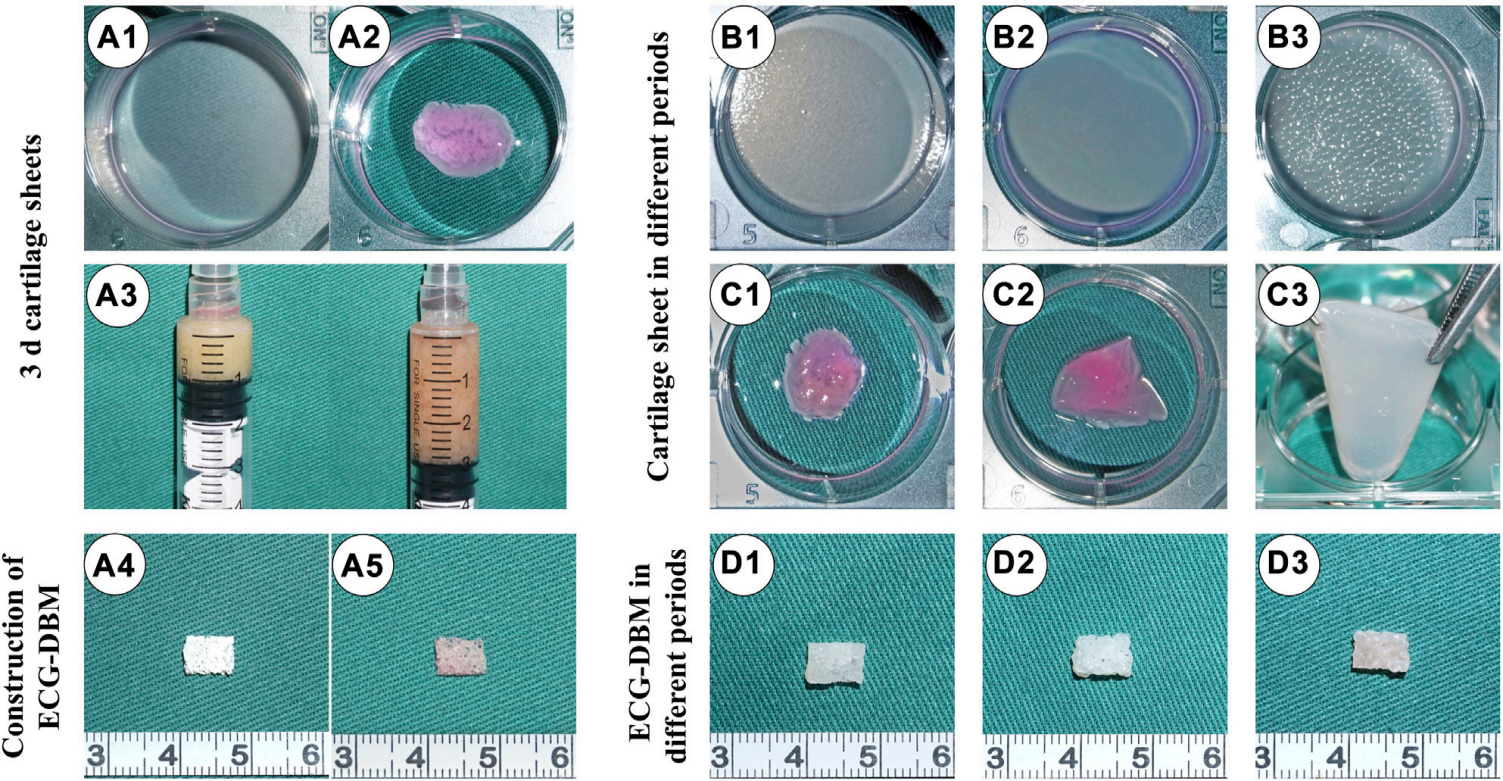
Preparation of ECG and fabrication of ECG-DBM. Gross **(A1)** and mature status **(A2)** images of a 3-d cartilage sheet. 1:2 dilution of 3-d cartilage gel **(A3)**. Gross views of the DBM scaffold **(A4)** and ECG-DBM **(A5)**. Gross **(B1)** and mature status **(C1)** images of a 4-d cartilage sheet. Gross **(B2)** and mature status **(C2)** images of a 7-d cartilage sheet. Gross **(B3)** and mature status **(C3)** images of a 14-d cartilage sheet. Gross images of ECG-DBM constructs after 4 days **(D1)**, 7 days **(D2)**, and 14 days **(D3)** of *in vitro* incubation.

After 3 days of *in vitro* culture, the ECG cartilage sheets exhibited colloidal properties (Figures A1,A2). The 3-day sheets were collected in a 5-mL syringe and mixed with a 1.5-fold volume of medium to prepare 3-d ECG (Figure 1A3). The 3-d ECG was then inoculated into the DBM scaffold (Figure 1A4) to construct the ECG-DBM complex (Figure 1A5). After further *in vitro* culture, it was noted that the 7-day cartilage sheets still exhibited colloidal properties (Figures 1B1,B2,C1,C2), while the 14-day cartilage sheets were relatively stiff and could not be collected directly (Figures 1B3,C3). In the *in vitro* culture of the ECG-DBM complex, the scaffold void gradually became filled with ECG and exhibited a more pronounced creamy-white cartilage-like tissue (Figures 1D1–D3).

### 3.2 Biocompatibility between ECG and DBM

**FIGURE 2:**
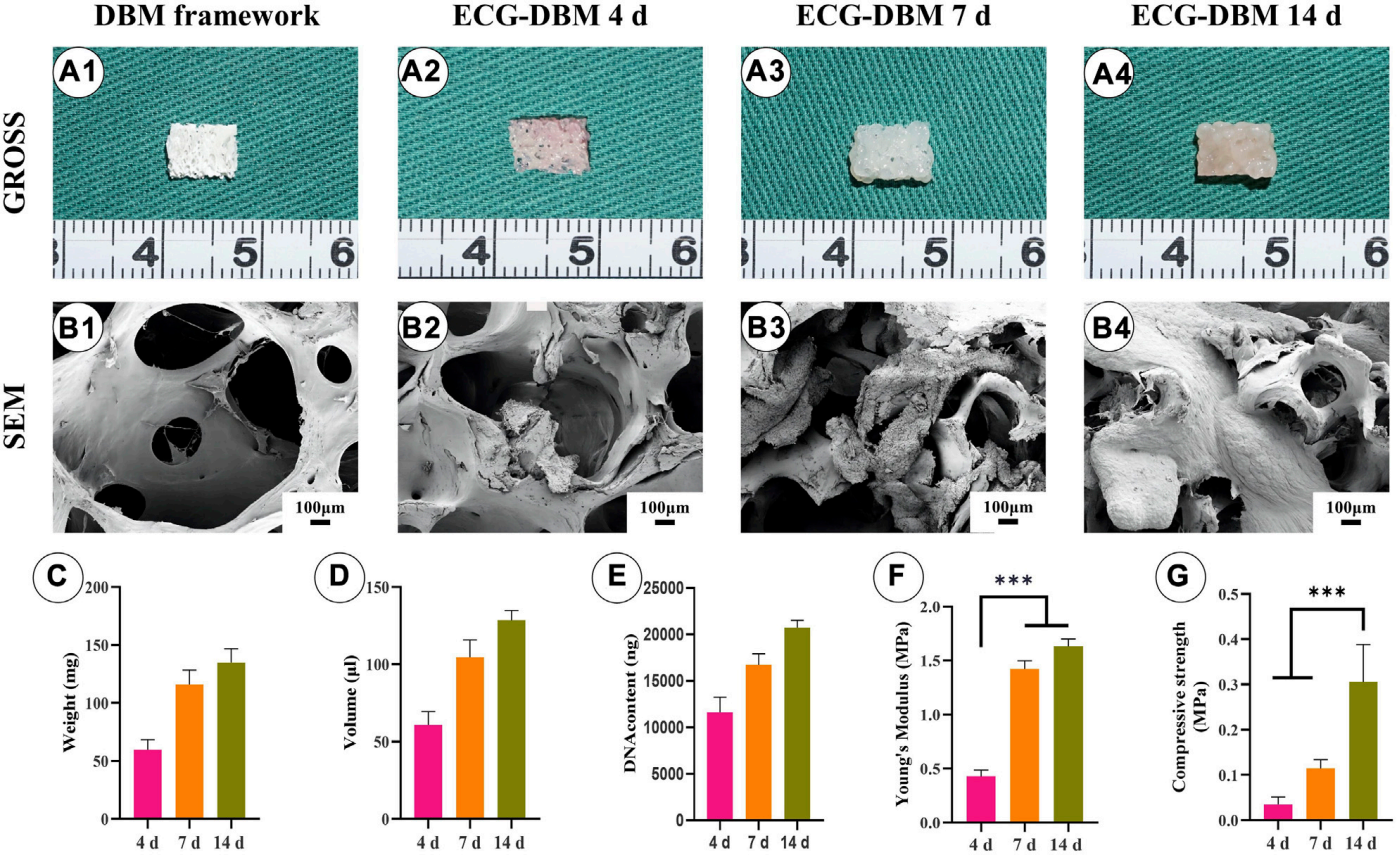
Evaluation of the biocompatibility of ECG and DBM. Gross **(A1)** and SEM **(B1)** images of the DBM framework. Gross **(A2)** and SEM **(B2)** images of the ECG-DBM constructs after culture *in vitro* for 4 days. Gross **(A3)** and SEM **(B3)** images of the ECG-DBM constructs after culture *in vitro* for 7 days. Gross **(A4)** and SEM **(B4)** images of the ECG-DBM constructs after *in vitro* culture for 14 days. The wet weight **(C)**, volume **(D)**, DNA content **(E)**, Young’s modulus **(F)**, and compressive strength **(G)** of ECG-DBM at different time points. ***p < 0.001.

The pore size of DBM was very large (800–1,200 μm) for direct cell inoculation (Figures 2A1,B1). However, 3-d ECG showed good biocompatibility with DBM and was able to stably adhere to it. On gross morphology observation, the ECG-DBM complex gradually matured with increasing time in culture, changing from a pink tissue with a rough surface to a typical creamy-white cartilage-like tissue with a smooth surface (Figures 2A2–A4). SEM observation confirmed that 3-d ECG was attached to the DBM scaffold. Almost all of the inoculated ECG was retained in the DBM (24-h ECG adhesion rate: 70%), and a large amount of cartilage extracellular matrix (ECM) was produced. The new cartilage ECM gradually filled the pores of the DBM scaffold as the culture time increased (Figures 2B2–B4). Quantitative analysis revealed that the volume, wet weight, and DNA content of the ECG-DBM complex steadily increased with increasing time in culture (Figures 2C,D,E). Taken together, these findings indicated good cytocompatibility of the DBM scaffold. The ECG-DBM complex exhibited excellent mechanical strength after 7 and 14 days of *in vitro* culture (Figures 2F,G), which should help the composite to maintain its shape *in vivo*. Therefore, this complex was selected as the composite material for subsequent experiments.

### 3.3 *In vitro* cartilage regeneration performance of the ECG-DBM complex

**FIGURE 3:**
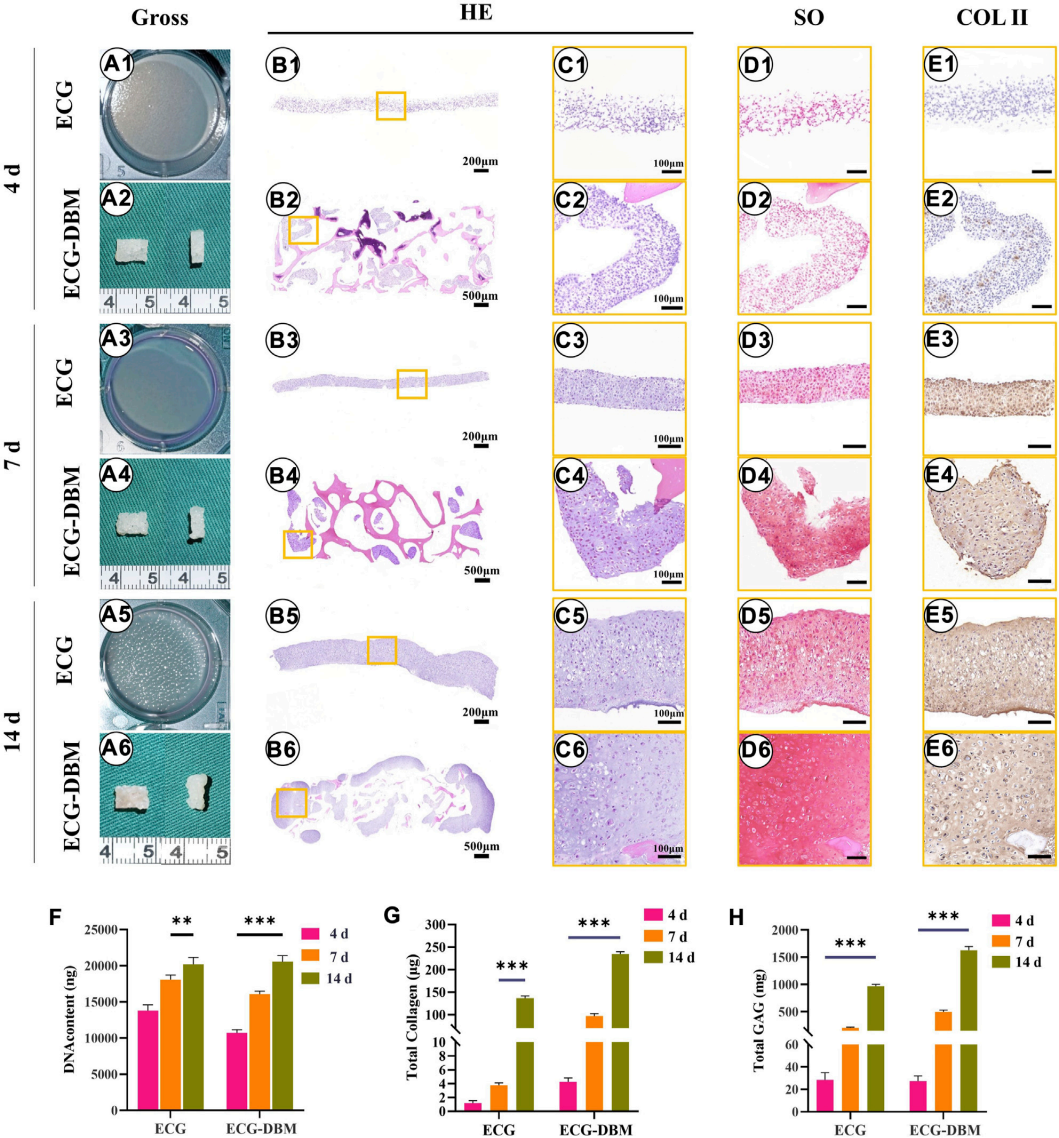
Assessment of the cartilage regeneration ability of ECG and ECG-DBM *in vitro*. Gross view **(A1–A6)**; H&E staining with different magnification **(B1–B6, C1–C6)**; safranin-O **(D1–D6)**, and immunohistochemical COL-2 **(E1–E6)** staining in ECG and ECG-DBM groups. Quantitative analysis of DNA content **(F)**, total collagen **(G)**, and GAG content **(H)** in the ECG and ECG-DBM groups. **p < 0.01; ***p < 0.001.

To verify the effectiveness of the ECG-DBM complex for cartilage regeneration, we performed *in vitro* chondrogenic induction cultures of ECG. The aforementioned ECG-DBM complex and the same culture conditions were used for quantitative analysis of the differences in chondrogenesis. Gross morphology observation showed that the ECG surface gradually became uneven with increasing time in culture. At the same time, the ECG-DBM complex was able to maintain its original shape. Furthermore, the surface pores gradually became filled with tissue. The color slowly changed to the typical creamy-white color of cartilage (Figures 3A1–A6). Histologically, weak positive staining for COL-2 was observed in the ECG-DBM group (Figures 3B1–E1, B2–E2). After 7 days of culture, both groups successfully formed cartilage-like tissue with a pinkish-white appearance (Figures 3A3, A4). However, HE staining, at this time point, showed that both groups had inconspicuous cartilage traps (Figures 3B3–C3, B4–C4). The staining results indicated that the ECG group was negative. However, the ECG-DBM group showed scattered positivity (Figures 3D3, D4). COL-2 staining was positive in both groups (Figures 3E3, E4). The cartilage tissue was more mature after 14 days, with typical cartilage lacunae observed on histological examination in both groups (Figures 3B5–C5, B6–C6). The SO staining results showed scattered positivity in the ECG group, while the ECG-DBM group was strongly positive (Figures 3D5, D6). COL-2 staining was strongly positive in both groups (Figures 3E5, E6).

Further quantitative analyses revealed that the DNA, total collagen, and GAG contents in the regenerated cartilage tissues increased significantly in both groups with increasing time in culture (Figures 3F–H). The contents in the ECG-DBM group remained higher than those in the ECG group after removing any influence of the collagen and GAG contents in the DBM scaffold itself. Taken together, these results indicate that the ECG-DBM composite has good *in vitro* cartilage regeneration ability and is significantly better than the ECG cartilage sheet alone.

### 3.4 Cartilage regeneration performance of the ECG-DBM composite *in vitro* and *in vivo*

After confirming that the ECG-DBM complex exhibited better cartilage regeneration ability than the ECG cartilage sheet *in vitro*, the *in vivo* cartilage regeneration ability of the ECG-DBM complex was investigated after subcutaneous implantation into nude mice, following culture for different times *in vitro*.

**FIGURE 4:**
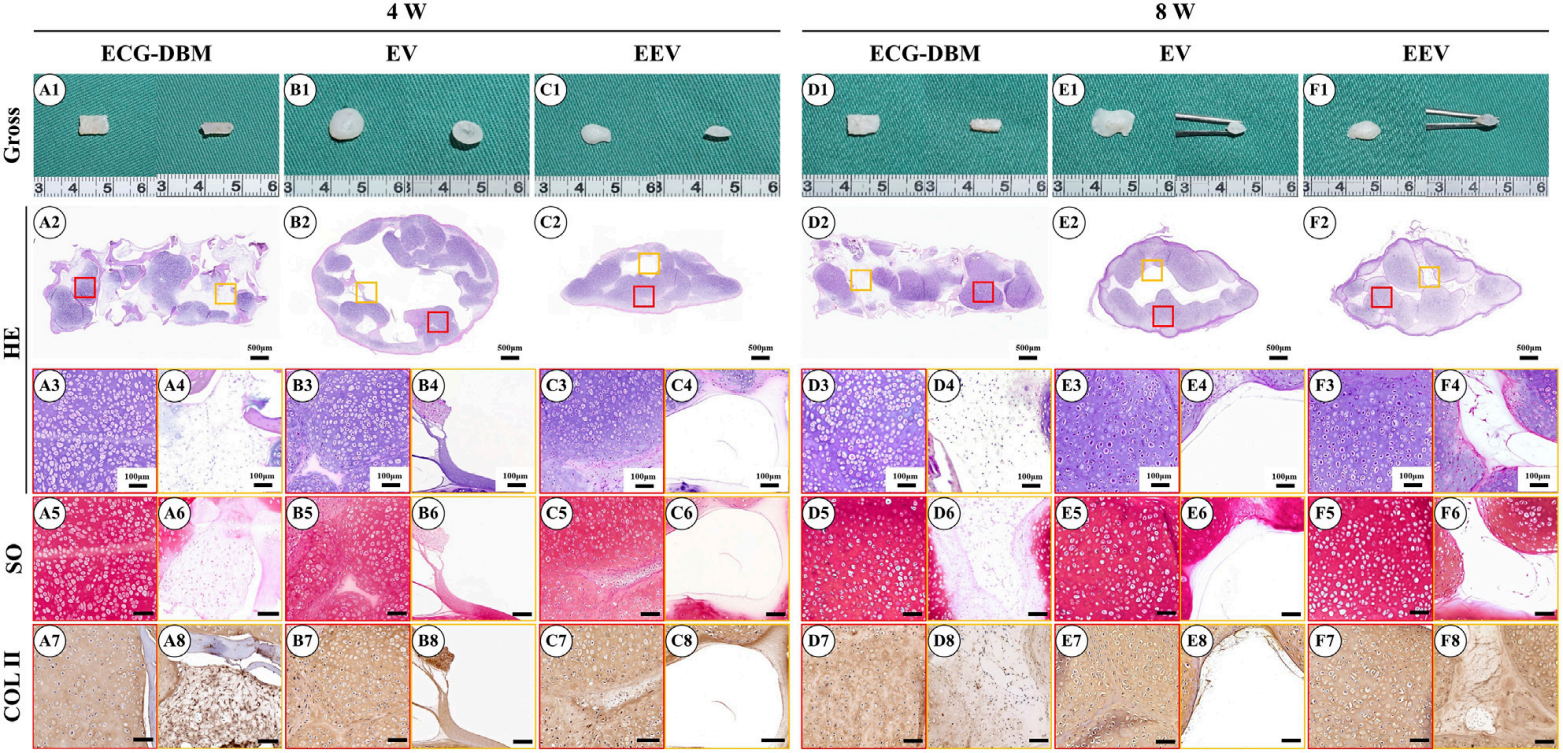
Assessment of the cartilage regeneration ability in nude mice of ECG and ECG-DBM groups after 7 days of *in vitro* culture. Gross observations, HE, safranin-O, and type II collagen immunohistochemical staining were performed on ECG-DBM **(A1–A8, D1–D8)**, equivalent ECG volume **(B1–B8, E1–E8),** and equivalent ECG content **(C1–C8, F1–F8)**. Red boxes represent areas of mature cartilage-like tissue, and orange boxes represent immature cartilage-like tissue or blank areas.

After 7 days of *in vitro* culture and implantation under the skin of nude mice, gradually maturing regenerated cartilage was observed. Typical creamy-white cartilage-like tissue was formed in all mice after 8 weeks (Figures 4A1–F1). However, only the samples in the ECG-DBM group maintained their original shape and size with a relatively regular rectangular shape. The ECG group showed an irregular shape due to the lack of a supporting frame. The histological examination confirmed that the volume and shape were maintained in the ECG-DBM group. The center part of the regenerated cartilage in the ECG group was significantly immature (Figures 4A2–F2), and this was most evident in the EV group at 4 weeks. In the control group, the immature part in the center of the regenerated tissue was significantly reduced at 8 weeks compared with 4 weeks. Cartilage-specific staining showed that all groups formed mature cartilage-like tissue with a typical lacuna structure and abundant cartilage-specific ECM deposition with strong positive staining for SO and COL-2 (Figures 4A3–F8).

**FIGURE 5:**
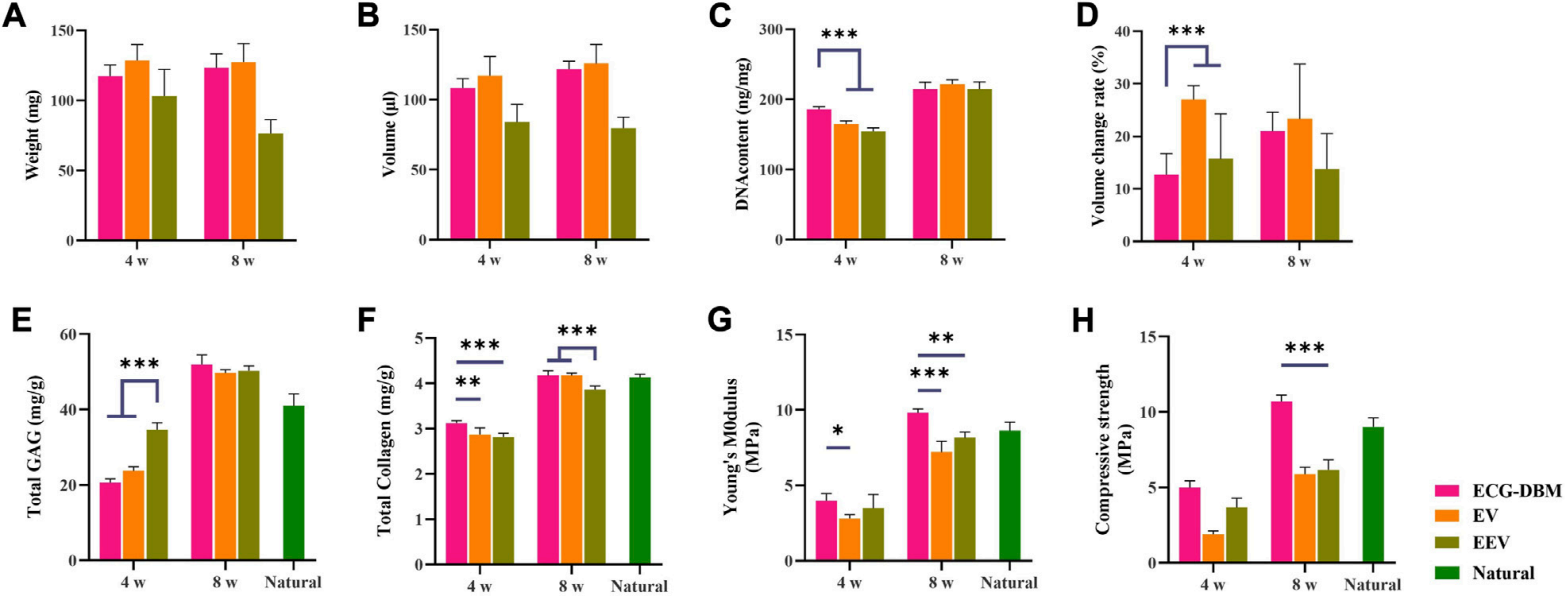
After 4 and 8 weeks of subcutaneous implantation in nude mice, the regenerated cartilage was quantitatively evaluated in ECG-DBM, EV, and EEV: **(A)** wet weight, **(B)** volume, **(C)** DNA content, **(D)** volume change rate, **(E)** total glycosaminoglycan (GAG) content, **(F)** total collagen content, **(G)** Young’s modulus, and **(H)** compressive strength. Statistical significance: **p < 0.01 and ***p < 0.001.

Further quantitative analyses revealed that the GAG content was significantly higher in the EEV group at 4 weeks (Figure 5E). All three groups had regenerated cartilage tissue of comparable quality at 8 weeks. The total collagen content showed the same trends for the differences at 4 and 8 weeks. The total collagen content at 8 weeks was comparable to that in normal cartilage tissue (Figure 5F). The analyses of the biomechanical properties (compressive modulus and compressive strength) showed that the regenerated cartilage in the ECG-DBM group was superior to that in the ECG group at both 4 and 8 weeks (Figures 5G, H).

### 3.5 Assessment of the ECG-DBM composite after *in vitro* culture for 14 days

**FIGURE 6:**
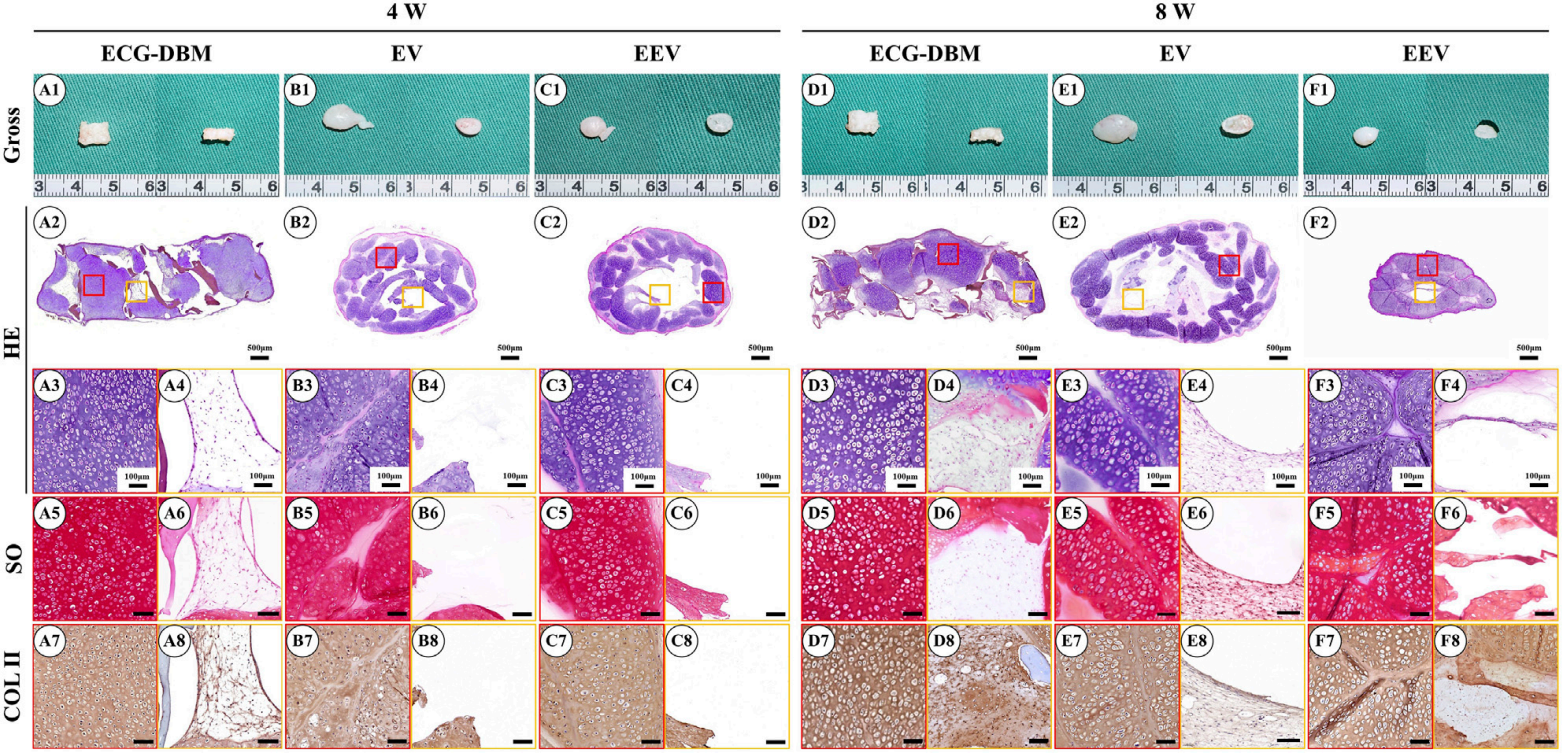
Gross observation and histological examination of regenerated cartilage after 14 days of *in vitro* culture of engineered cartilage gel (ECG) implanted in nude mice for 4 and 8 weeks. Gross observations, HE, safranin-O, and type II collagen immunohistochemical staining were performed on ECG-DBM **(A1–A8, D1–D8)**, EV **(B1–B8, E1–E8),** and EEV **(C1–C8, F1–F8)**. Red boxes represent areas of mature cartilage-like tissue, and orange boxes represent immature cartilage-like tissue or blank areas.

Following subcutaneous implantation into nude mice after 14 days of *in vitro* culture, all groups showed the same maturation trend, as the groups with subcutaneous implantation after 7 days of *in vitro* culture. Specifically, gross morphology observation revealed that the ECG-DBM and ECG groups both formed mature cartilage-like tissue in the outer areas of the samples at 8 weeks (Figures 6A1–F1). However, the tissue in the ECG-DBM group was more homogeneous, and its volume and shape were well maintained with a relatively regular rectangular shape. There were relatively fewer intermediate cavity areas in the ECG group. However, a decrease in the volume was noted in the EEV group at 8 weeks. Histological examination confirmed that the samples from the ECG-DBM group were more homogeneous and that some immature cartilage and fibrous-like tissues were present in the center of the ECG group samples (Figures 6A2–F2). No significant differences in cartilage-specific staining were observed, indicating that all groups formed mature cartilage-like tissue *in vivo* with a typical lacuna structure and strong positive staining for SO and COL-2 (Figures 6A3–F8).

**FIGURE 7:**
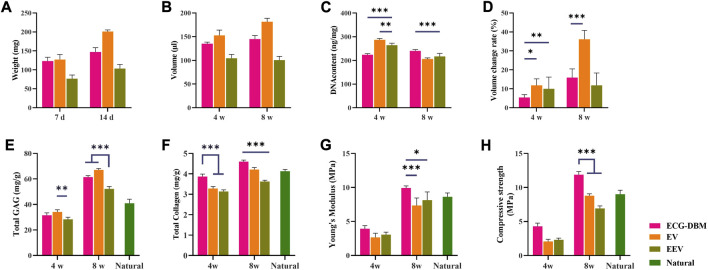
Quantitative evaluation of engineered cartilage gel-decalcified bone matrix (ECG-DBM) constructs cultured *in vitro* for 14 days. After 4 and 8 weeks of subcutaneous implantation in nude mice, the regenerated cartilage was quantitatively evaluated in ECG-DBM, EV, and EEV: **(A)** wet weight, **(B)** volume, **(C)** DNA content, **(D)** volume change rate, **(E)** total glycosaminoglycan (GAG) content, **(F)** total collagen content, **(G)** Young’s modulus, and **(H)** compressive strength. Statistical significance: *p < 0.05, **p < 0.01, and ***p < 0.001.

The quantitative analyses showed the same trends for the differences in the GAG content, total collagen content, and Young’s modulus at 4 and 8 weeks, with no significant difference in the GAG content between the ECG-DBM and EV groups at 8 weeks, although both groups were better than the EEV group (Figure 7E). The results for the total collagen content, Young’s modulus, and compressive strength showed that the ECG-DBM group was superior to both the EV and EEV groups, and was also superior to normal tissue (Figures 7F–H).

### 3.6 *In vivo* cartilage regeneration effects of the ECG-DBM complex after *in vitro* culture for different times

**FIGURE 8:**
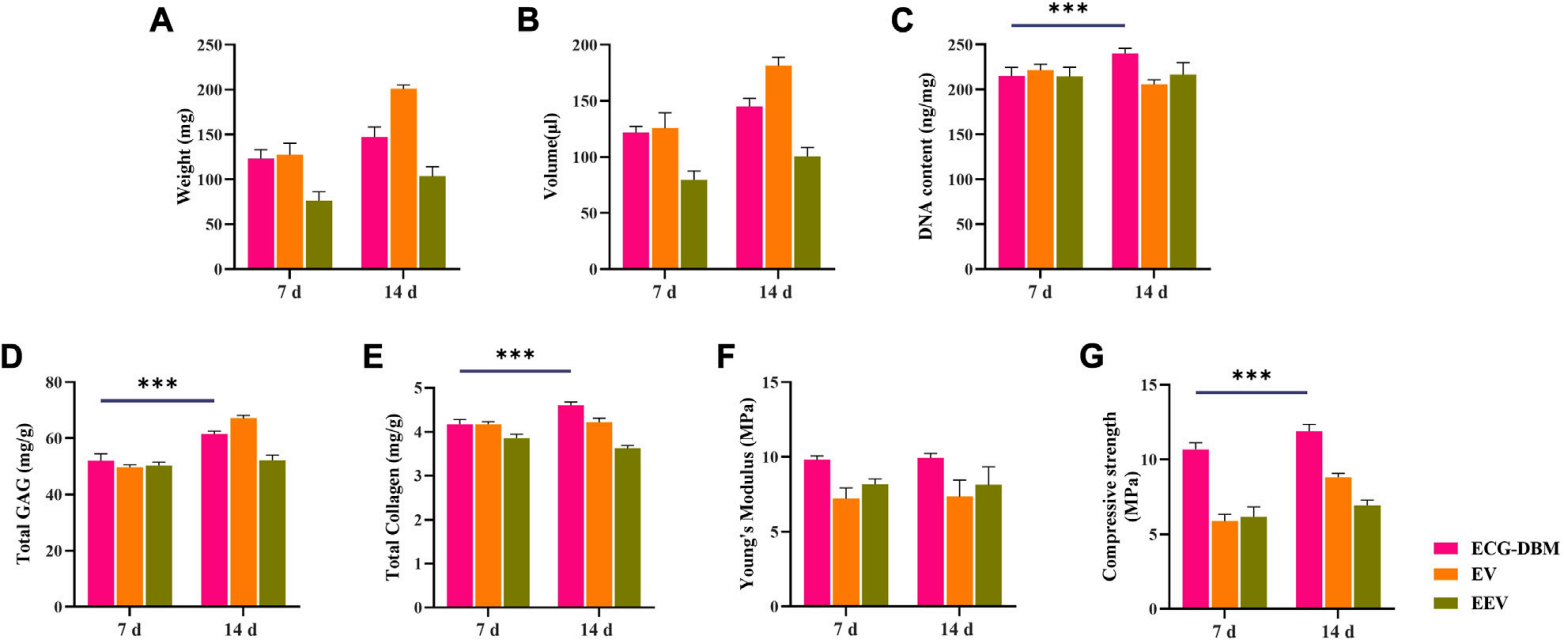
Quantitative analysis of ECG-DBM complexes cultured *in vitro* for 7 and 14 days, *in vitro* and after 8 weeks of subcutaneous implantation in nude mice comparing **(A)** wet weight, **(B)** volume, **(C)** DNA content, **(D)** total glycosaminoglycan (GAG) content, **(E)** total collagen content, **(F)** Young’s modulus, and **(H)** compressive strength. ***p < 0.001.

To clarify the optimal time for *in vivo* implantation, we further compared the findings for the ECG-DBM complex after *in vitro* culture for 7 and 14 days to assess the ability for *in vivo* cartilage regeneration. The quantitative analyses revealed higher values for the ECG-DBM group than the EV and EEV groups at 8 weeks after implantation (Figure 5; Figure 7). These results indicated that the ECG-DBM group exhibited better cartilage regeneration, more mature cartilage lacuna, and more abundant ECM secretion. On further quantitative analyses of the ECG-DBM complex at 7 days *versus* 14 days, the regenerated cartilage tissue in the 14-day group had comparable mechanical strength *in vivo* to the 7-day group (Figure 8F) but superior compressive strength (Figure 8G). The GAG and total collagen contents in the 14-day group were also superior to those in the 7-day group (Figures 8D, E). Histological staining confirmed that the ECG-DBM complex in the 14-day group regenerated more homogeneous cartilage tissue at 8 weeks (Figure 4A2; Figure 6A2). These findings indicate that an appropriate prolongation of the *in vitro* culture time facilitates more homogeneous cartilage regeneration. Taken together, the present results suggest that the cartilage regeneration strategy involving the ECG-DBM complex shows good potential for translation into clinical practice.

## 4 Discussion

The limitation of the thickness of regenerated cartilage remains a significant challenge for engineering of cartilage with a specific size and suitable shape for clinical application (Liau et al., 2021; Wang et al., 2022). The present study showed that the ECG-DBM complex produced better chondrogenic effects *in vitro* and *in vivo* after subcutaneous implantation into nude mice. The ECG-DBM complex and ECG cartilage sheets showed considerable chondrogenic ability, forming cartilage-like tissue with a certain thickness and a progressively mature cartilage appearance. When chondrogenesis was examined *in vivo*, all groups regenerated high-quality cartilage tissue. Notably, although the EV group exhibited comparable chondrogenic capacity to the ECG-DBM group, the internal cavities and poor mechanical properties in the EV group were detrimental for clinical application (Olubamiji et al., 2016; Krishnan and Grodzinsky, 2018). Meanwhile, the EEV group showed significant resorption, which significantly limited the clinical application for specific morphological cartilage defects. It is also noteworthy that an appropriate extension of the *in vitro* culture time helped improve the poor internal cartilage formation. The present study suggests that the ECG-DBM complex can take advantage of both the chondrogenesis of ECG and the 3D morphology maintenance of DBM to achieve better regeneration of the specific morphology and volume of cartilage tissue, thereby broadening the clinical applications of ECG.

Cartilage sheet technology is a promising strategy for cartilage regeneration, but the uncontrollable shape and poor mechanical strength significantly hinder its clinical application. DBM has excellent biocompatibility and structural stability, and is a promising biomaterial widely used in tissue engineering (Mattioli-Belmonte et al., 2019; Chen et al., 2021a; Hao et al., 2022). Our previous study established a new model for cartilage regeneration using a steel-reinforced concrete concept by inoculating ECG into a DBM scaffold. The present study verified the feasibility of the previous model and optimized the culture cycle for the ECG-DBM complex before systematically comparing the *in vitro* cartilage regeneration capacities of ECG in the simple cartilage sheet state and the ECG-DBM complex state. The advantages for cartilage regeneration afforded by the ECG-DBM technology were further clarified by comparing the cartilage regeneration effects of the ECG-DBM complex with those of EV and EEV. The findings showed that the ECG-DBM complex regenerated higher-quality engineered cartilage with better shape maintenance and cartilage regeneration efficiency than ECG. We consider that the better shape maintenance of the ECG-DBM complex was mainly conferred through the mechanical support for the structure provided by DBM and that this feature can meet the clinical demand for different volumes and morphologies of regenerated cartilage. Meanwhile, the more efficient cartilage regeneration exhibited by the ECG-DBM complex mainly arises through the ability of ECG to secrete cartilage ECM more efficiently than chondrocytes alone after *in vitro* culture.

The present study further showed that the ECG-DBM complex taken at later time points of *in vitro* culture regenerated cartilage tissue that was more homogeneous and had smaller internal cavities. Both the EV and EEV groups showed varying degrees of cavities within the regenerated cartilage tissue. These cavities may be caused by the 3D porous sponge-like structure of DBM with a larger pore size (800–1,200 μm) and higher porosity than conventional cartilage regeneration scaffolds (pore size: 80–120 μm) and the feature of interconnected pore structures (Tuan et al., 2013; Theodoridis et al., 2022; Lin et al., 2023; Xue et al., 2023). DBM can provide a broad inner surface area and ample space for ECG and sufficient space for the nutrient exchange needed by the tissue. The large pore size also allows cartilage matrix formation at the periphery of the tissue mass while still leaving space for a nutrient exchange channel to facilitate internal tissue regeneration by permitting uniform secretion of cartilage matrix components throughout the ECG-DBM complex.

Although the present study further validated the feasibility and advantages of cartilage regeneration with a steel-reinforced concrete model, the findings were only based on subcutaneous implantation in nude mice. In future studies, we will further explore the feasibility of creating precisely shaped cartilage constructs using a steel-reinforced concrete model to repair cartilage defects in animals with long-term immunity.

## 5 Conclusion

The present findings confirmed the feasibility of the ECG-DBM complex strategy for cartilage regeneration, verified the production of an ECG-DBM composite material with higher cartilage regeneration efficiency, and improved the homogeneity of the regenerated cartilage. Compared with conventional ECG cartilage sheets, the ECG-DBM complex had a more controllable shape, good morphological retention, moderate mechanical strength, and high cartilage regeneration efficiency. This study provides some possibilities and guidance for the application of DBM in cartilage tissue engineering.

## Data Availability

The original contributions presented in the study are included in the article/Supplementary Material; further inquiries can be directed to the corresponding authors.
